# Aristolactams and Alkamides of *Aristolochia gigantea*

**DOI:** 10.3390/molecules15129462

**Published:** 2010-12-21

**Authors:** Juliana C. Holzbach, Lucia M. X. Lopes

**Affiliations:** Instituto de Química, Universidade Estadual Paulista, CP 355, 14801-970 Araraquara-SP, Brazil

**Keywords:** *Aristolochia gigantea*, Aristolochiaceae, aristolactams, alkamides

## Abstract

A new aristolactam, aristolactam 9-*O*-β-D-glucopyranosyl-(1→2)-β-D-glucoside, and two alkamides, *N-cis-* and *N-trans-p-*coumaroyl-3-*O*-methyldopamine, were isolated from stems of *Aristolochia gigantea*, together with the known compounds allantoin, *E*-nerolidol, β-sitosterol, (+)-kobusin, (+)-eudesmin, *trans*-*N*-feruloyltyramine, *trans*-*N*-coumaroyltyramine, *trans*-*N*-feruloyl-3-*O*-methyldopamine, aristolactam Ia-*N*-β-D-glucoside, aristolactam Ia 8-β-D-glucoside, aristolactam IIIa, and magnoflorine. Their structures were determined by spectroscopic analyses.

## 1. Introduction

The Aristolochiaceae family consists of 450 to 600 species, among which more than 200 have been at least partially studied [1]. Most of these studies have focused on a characteristic group of phenanthrenic compounds, which includes the aristolochic acids (AAs) and the aristolactams (ALs), the former of which occur mainly in species of the genus *Aristolochia*.

In some European countries and until recently in Brazil, *Aristolochia* herbs have been used in weight-loss regimens. The clinical application of aristolochic acid (AA) has been limited due to its severe nephrotoxic activity. Recent studies have revealed that AA-I can cause direct damage to renal tubular cells, and its carcinigenicity is associated with the formation of promutagenic AA-DNA adducts [2,3]. The cytotoxic potency of AL-I is higher than that of AA-I, and the cytotoxic effects of these molecules are mediated through the induction of apoptosis in a caspase 3-dependent pathway [3]. Consequently many countries have now banned the use of herbs containing AAs and ALs and the US Food and Drug Administration has banned the sale of all products that contain AAs and ALs in their formulations [4].

Recently, the aristolactams have received much attention due to an interesting array of biological properties, including anti-inflammatory, antiplatelet, antimycobacterial, and neuro-protective activities [5]. Naturally occurring aristolactams and several synthetic aristolactam derivatives have been shown to have potent antitumor activities against a broad array of human cancer cell lines. Several aristolactams which may possess postcoital antifertility activity have been isolated from *Aristolochia indica*. In addition, neurological disorders, especially Parkinson’s disease, have been treated by administration of the aristolactam taliscanine [6]. Brazilian *Aristolochia* species, including *Aristolochia gigantea*, have been used in traditional medicine as abortifacients and in the treatment of wounds and skin diseases [7].

*Aristolochia gigantea* develops a strong system of subterranean stems and roots (tuberous or rhizomatous roots). α-Phellandrene (60.9%) and linalool (16.6%) are the major constituents of the essential oil obtained from these plant parts [8], whereas germacrene D and γ-elemene are the most abundant compounds in the leaf oils. *trans*-Nerolidol and geraniol are the major constituents in the stem and flower oils, respectively [9]. Previous studies on the leaves of this plant have also led to the isolation of allantoin and sitosterol [7], which are also found in significant quantities in other Aristolochiaceae species. In addition, salsolinol, higenamine, and pinitol have been isolated together with several bisbenzylisoquinolinic and 8-benzylberberinic alkaloids from *A. gigantea*. These latter compounds have an unusual carbon skeleton [7,10,11]. As part of our continuing studies on the Aristolochiaceae family, we report here the isolation and structural elucidation of aristolactams and alkamides, among other compounds, from aerial and ground (rhizomes) stems of *A. gigantea*.

## 2. Results and Discussion

**Figure 1:**
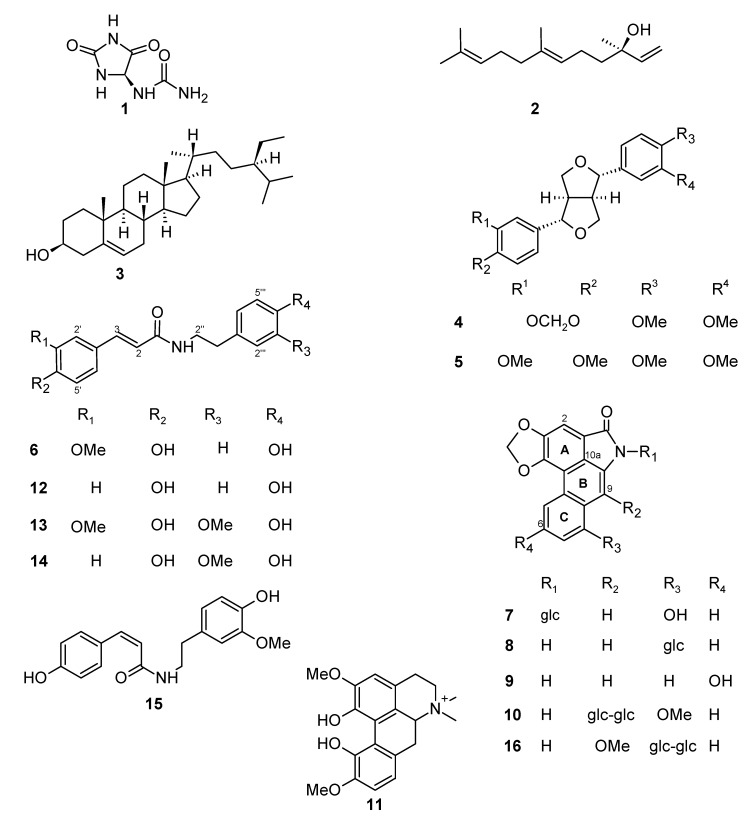
Chemical structures of compounds **1**–**16**.

Compounds **1**–**15** (Figure 1) were isolated by chromatography and partition procedures from the ethanol extracts of the stems and analyzed by spectrometric methods (IR, UV, MS, 1D- and 2D-NMR).Phytochemical studies on the ethanol extract from rhizomes of *A. gigantea* led to the isolation of 10 known compounds: allantoin (**1**) [12], *E*-nerolidol (**2**) [13,14], β-sitosterol (**3**) [15,16], (+)-kobusin (**4**) [17], (+)-eudesmin (**5**) [17], *trans*-*N*-feruloyltyramine (**6**) [18], aristolactam Ia *N*-β-D-glucoside (**7**) [19], aristolactam Ia 8-β-D-glucoside (**8**) [20,21], aristolactam IIIa (**9**) [22], and magnoflorine (**11**) [23], together with a new aristolactam (**10**). In addition, four known compounds, (+)-kobusin (**4**), *trans*-*N*-feruloyltyramine (**6**), *trans*-*N*-coumaroyltyramine (**12**) [18], and *trans*-*N*-feruloyl-3-*O*-methyldopamine (**13**) [24], and a mixture of *cis* and *trans* new alkamides (**14** + **15**) were obtained from the aerial stems. The structures of the known compounds were determined by analyses of their physical and spectroscopic data and comparison of these data to those reported in the literature and to those of authentic samples available in our laboratory, which were previously isolated from *Aristolochia* spp.

A molecular formula of C_29_H_31_O_15_N was determined for compound **10** based on its HRMS spectra, which showed *quasi*-molecular ions at *m/z* 632.1614 [M – H]^−^. The IR spectrum of compound **10** showed characteristic absorption bands of a lactam group at 1,654 cm^−1^ and hydroxyl groups at 3,442 and 1,088 cm^−1^. The DEPT and ^13^C-NMR spectra of **10** (Table 1) showed signals for 14 aromatic carbons, and acyl (δ_C_ 167.4), methylenedioxy (δ_C_ 103.0), and methoxyl (δ_C_ 56.0) groups. 

**Figure 2:**
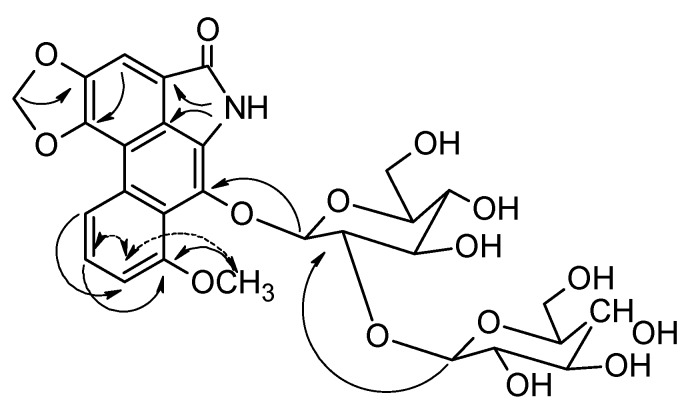
Selected *g*HMBC (→) correlations and nOe (↔) interactions for aristolactam **10**.

The ^1^H-NMR spectrum showed signals characteristic of CONH at δ 10.18 and only four aromatic hydrogens at δ 8.26 (dd, *J* = 8.5, 1.0), 7.56 (dd, *J* = 8.5, 8.0), 7.23 (dd, *J* = 8.0, 1.0), and 7.65 (s). In addition, signals for carbons and hydrogens for a diglycosyl were observed. These data suggested that compound **10** was an aristolactam. ^1^H-^1^H COSY and 1D-TOCSY experiments allowed us to determine that the glycosyl units were β-glucosyl-β-glucosyl (1→2). Furthermore, the negative ESI-MS/MS of the ions at *m/z* 632.1 gave rise to ions at *m/z* 308.1 and 469.9 that suggested C9-O and O-C1'' fragmentations, respectively. The substituent positions on the aristolactam structure were assigned with the help of *g*HMBC experiments (Figure 2). These experiments showed correlations between C-9 (δ_C_ 132.6) and H-1' (δ_H_ 5.08); C-3(δ_C_ 148.2) and CH_2_O_2_ (δ_H_ 6.46); C-8 (δ_C_ 157.2) and OCH_3_ (δ_H_ 3.94) and H-6 (δ_H_ 7.56), as well as between C-2′ (δ_C_ 81.0) and H-1″ (δ_H_ 4.65). 

The ^1^H- and ^13^C-NMR, IR, and UV spectroscopic data of **10** were very similar to those reported in the literature for triangularine (**16**; Figure 1) [25], the main difference being due to interchange of the substituents at C-8 and C-9. The location of the methoxyl group at C-9 in **10** was corroborated by *g*NOESY experiments that showed an interaction between CH_3_O and H-7. This new aristolactam was named aristolactam 9-*O*-β-D-glucopyranosyl-(1→2)-β-D-glucoside.

**Table 1:** NMR data for compound **10**.^ a^

Position	*δ*_H_	*δ*_C_	Position	*δ*_H_	*δ*_C_
1		119.1	OCH_2_O	6.46 *s*	103.0
2	7.65 *s*	105.7	OCH_3_	3.94 *s*	56.0
3		148.2	1′	5.08 *d* (6.5)	103.0
4		146.9	2′	3.92 *dd* (8.5, 6.5)	81.0
4a		109.0	3′	3.54 *t* (8.5)	75.8
4b		127.9	4′	3.47 *t* (8.5)	69.4
5	8.26 *dd* (8.5, 1.0)	118.4	5′	3.18 *m*	76.9
6	7.56 *dd* (8.0, 8.5)	126.1	6′α, 6′β	3.8 − 3.6 *m*	60.5
7	7.23 *dd* (8.0, 1.0)	110.8	1″	4.65 *d* (7.5)	102.4
8		157.2	2″	3.06 *dd* (7.5, 8.5)	74.1
8a		120.0	3″	3.10 *t* (8.5)	76.1
9		132.6	4″	3.17 *t* (8.5)	69.3
10		^b^	5″	2.99 *ddd* (8.5, 4.7, 2.5)	76.4
10a		124.4	6″α, 6″β	3.35 *m*	60.3
				3.8 − 3.6 *m*	
CO		167.4	NH	10.18 *s*	

^a ^The ^1^H- and ^13^C-NMR data were assigned with the assistance of *g*HMQC, *g*HMBC, and ^1^H-^1^H COSY experiments (11.7 T); recorded in DMSO-*d_6_*; *J* in Hz; ^b ^Signal not observed.

**Figure 3:**
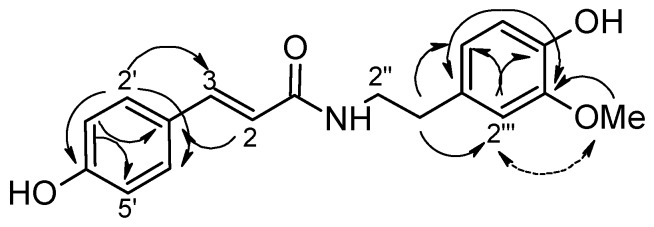
Select *g*HMBC (→) correlations and nOe (↔) interactions for alkamide **14**.

The ^1^H- and ^13^C-NMR spectra of **14 **+ **15** (Table 2) were very similar to those of **13**, except for the absence of a methoxyl group at C-3' in **13**, and suggested that it consisted of *cis*- and *trans-*alkamides with *p*-disubstituted and trisubstituted aromatic rings. The molecular formula (C_18_H_19_O_4_N) deduced from the HRMS spectra was also consistent with the lack of an OCH_3_ substituent. Based on the integration of the signals corresponding to the olefinic hydrogens [*cis*: δ_H-2_ 5.75 (d, *J* = 13.0) and δ_H-3_ 6.48 (d, *J* = 13.0); *trans*: δ_H-2_ 6.38 (d, *J* = 15.5) and δ_H-3_ 7.28 (d, *J* = 15.5)] it was possible to determine that the isolated mixture was in a 1:2 *cis*/*trans* proportion. Although *cis* and *trans* isomers can isomerize under UV light, both alkamide isomers may be natural compounds [26,27]. To assign with confidence all of the chemical shifts for carbons and hydrogens in the structures, this mixture was exposed to daylight for four hours. The subsequent ^1^H-NMR spectrum revealed that the *cis*/*trans* proportion had changed to 2:1. The linkage of the methoxyl group to C-3''' was established based on the observation of a correlation between this carbon and the methoxyl hydrogens by *g*HMBC experiments, as well as by the spatial interactions of methoxyl hydrogens with H-2''', as observed by 1D-NOESY experiments (Figure 3). Correlations observed by *g*HMBC experiments also assisted to determine the carbon skeleton. These alkamides **14** and **15 **were named *N-cis-* and *N-trans-p-*coumaroyl-3-*O*-methyldopamine, respectively.

**Table 2:** NMR data for compounds **14**+**15**.^ a^

Position	14 *δ*_H_	15 *δ*_H_
2	6.38 *d* (15.5)	5.75 *d* (13.0)
3	7.28 *d* (15.5)	6.48 *d* (13.0)
2′, 6′	7.36 *d* (8.5)	7.56 *d* (8.5)
3′, 5′	6.76 *d* (8.5)	6.68 *d* (8.5)
2′	3.34 *m*^ b^	3.34 *m*^ b^
3′	2.62 *t* (5.5)	2.62 *t* (5.5)
2′′′	6.75 *d* (2.0)	6.74 *d* (2.0)
5′′′	6.66 *d* (8.0)	6.66 *d* (8.0)
6′′′	6.59 *dd* (8.0, 2.0)	6.58 *dd* (8.0, 2.0)
OCH_3_	3.72 *s*	3.71 *s*
NH	8.00 *t* (5.5)	7.98 *t* (5.5)

^a ^Recorded in DMSO-*d_6_*, 500 MHz, *J* in Hz; ^b^ Signals assigned with the assistance of ^1^H-^1^H COSY experiments.

Allantoin (**1**) is a product of purine metabolism and is widely distributed in biological systems. It has been isolated from marine sponges, animals, and numerous plants, including *Aristolochia* species. It is used as an anti-inflammatory, antipsoriatic (disputed), and topical vulnerary agent [28]. Allantoin is one of the best-known wound-healing agents, and exerts keratolytic and astringent effects and stimulates new tissue formation [29]. Other well-known compounds that were isolated from *A. gigantea* include *E*-nerolidol, which has been shown to possess larvicidal activity against *Aedes aegypti* [30] and antifungal activity against *Microsporum gypseum* [31], and magnoflorine, which exhibits insecticidal activity against *Spodoptera frugiperda* [32], among others activities [33].

## 3. Experimental

### 3.1. General

One-dimensional (^1^H, ^13^C, DEPT, and *g*NOESY) and two-dimensional (^1^H–^1^H *g*COSY, *g*NOESY, *g*HMQC, and^ 1^H–^13^C *g*HMBC) NMR experiments were performed on a Varian INOVA 500 spectrometer (11.7 T) at 500 MHz (^1^H) and 126 MHz (^13^C), using deuterated solvents (CDCl_3, _DMSO-*d*_6_) (99.98% D) as an internal standard for ^13^C-NMR chemical shifts, and residual solvent as an internal standard for ^1^H NMR. δ values are reported relative to TMS. Mass spectra (ESI-MS and ESI-MS/MS) were obtained on a Thermo LCQ, and flow injection into the electrospray source was used for ESI-MS. High-resolution mass spectra (HRMS) were obtained on a Bruker Daltonics MicroTOF Ic (ESI-TOFMS). IR spectra were obtained on a Perkin Elmer FT-IR Spectrum 2000 spectrometer using KBr discs. Optical rotations were measured on a Perkin–Elmer 341-LC polarimeter. Ultraviolet (UV) absorptions were measured on a Perkin–Elmer UV–vis Lambda 14P diode array spectrophotometer. HPLC analyses were performed using a Shimadzu liquid chromatograph (SPD-10 Avp) equipped with UV–Vis and 341-LC polarimeter detectors. RP-18 columns were used (Varian, C18, with a particle size of 5 µm, 250 by 4.6 mm for analytical analysis and 250 by 20 mm for semi-preparative analysis), and chromatograms were acquired at 336 and 254 nm. Melting points were recorded on a Microquímica MQAPF-302 melting point apparatus and are uncorrected.

### 3.2. Plant material

The plant material was collected in Araraquara, SP, Brazil, in February, 2004, and identified as *Aristolochia gigantea* Mart. (Aristolochiaceae) by Dr. Lindolpho Capellari Júnior (Escola Superior de Agricultura “Luiz de Queiroz” (ESALQ), Piracicaba, SP, Brazil). A voucher specimen (ESA 88281) was deposited at the herbarium of the ESALQ, Piracicaba, SP, Brazil. The material was separated according to the plant parts and dried (*ca.* 45 °C). The stems were further separated into aerial stems and rhizomes.

### 3.3. Extraction and isolation of the chemical constituents

The rhizomes (433.6 g) and aerial stems (379.4 g) were ground and exhaustively extracted successively at room temperature with hexane, acetone, and ethanol. The residues were extracted with ethanol in a Soxhlet apparatus and the extracts were individually concentrated. A portion of the crude ethanol extract of rhizomes (8.10 g) was washed with CH_3_OH. Compound **1** (43.0 mg) was isolated from the insoluble fraction. The methanol-soluble fraction was subjected to CC (6.0 by 40.0 cm, silica gel 60H, 127.3 g, hexane/CH_3_OH gradient, 19:1 to 100% CH_3_OH) to give 25 fractions (*ca.* 125 mL each). Fractions 7, 9, 12, 14, and 15 gave **2** (23.3 mg), **3** (25.0 mg), **4** (409.2 mg), **5** (158.3 mg), and **6** (26.2 mg), respectively. Fraction 23 after HPLC [Varian RP C18 semi-preparative column, eluted with CH_3_OH–H_2_O + 0.5% NH_4_OH, 3:2, flow = 8 mL min^−1^; λ = 254 nm] gave **1** (16.5 mg) and **11** (17.5 mg). Fraction 21 (1.10 g) was subjected to RP CC (3.0 by 45.0 cm, silica gel C18, 43.5 g, CH_3_OH–H_2_O gradient 9:1 to 100% CH_3_OH) to give 17 subfractions (*ca.* 125 mL each). Subfractions 10 and 11 (98.4 mg) after HPLC [Varian RP C18 column, eluted with CH_3_OH–H_2_O + 0.5% NH_4_OH 3:2, flow = 8 mL min^−1^, λ = 254 nm] gave **7** (4.0 mg), **8** (9.6 mg), **9** (1.8 mg), and **10** (10.1 mg). The stem crude ethanol extract (10.0 g) was fractionated over Sephadex LH-20 (120.0 g, 2.5 by 95.0 cm, MeOH) to give 17 fractions. Fraction 6 (166 mg) was subjected to two HPLC runs [Varian RP C18 semi-preparative column, eluted with CH_3_OH–H_2_O 3:2, flow = 8 mL min^−1^, λ = 254 nm; followed by Varian RP C18 analytical column, eluted with CH_3_OH–H_2_O 2:3, flow = 0.8 mL min^−1^, λ = 254 nm] to give **4** (2.0 mg), **12** (2.0 mg), **13** (1.5 mg), and **14+15** (1.0 mg)].

### 3.4. Spectral data

*Allantoin* (**1**). Yellow needles. mp 233–234 °C [lit. 232–235 °C] [12]. IR, ^1^H-NMR, and ^13^C-NMR data were consistent with those previously reported [12].

(−)*E-Nerolidol* (**2**). Yellow oil. 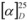 −17 ° (CHCl_3_, *c* 0.2) [lit. −12.5 ° (CHCl_3_, *c* 0.02)] [13]. ^1^H-NMR (CDCl_3_) δ 5.15 (1H, *dd*, *J* 17.5, 1.5 Hz, H-1α), 5.00 (1H, *dd*, *J* 10.5, 1.5 Hz, H-1β), 5.86 (1H, *dd*, *J* 17.5, 10.5 Hz, H-2), 1.54–1.51 (2H, *m*, H-4), 2.00–1.92 (6H, *m*, H-5, H-8, H-9), 5.08–5.01 (2H, *m*, H-6, H-10), 1.60 (3H, *s*, H-12), 1.22 (3H, *s*, H-15), 1.54 (6H, *s*, H-13, H-14). ^13^C-NMR data were consistent with those previously reported [14].

*β-Sitosterol* (**3**). Colorless crystals. 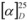 −15.2 ° (CHCl_3_, *c* 0.2) [lit. −26.1 ° (CHCl_3_, *c* 0.1)] [15]. ^13^C- NMR data were consistent with those previously reported [16].

(+)-*Kobusin* (**4**). Yellow solid. 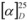 +51.4 ° (CHCl_3_, *c* 0.21) [lit. +58.0 ° (CHCl_3_, *c* 0.03)] [17]. ^1^H- NMR (CDCl_3_) δ 6.83 (1H, *d*, *J* 2.0 Hz, H-2), 6.78 (1H, *d*, *J* 2.0 Hz, H-2′), 6.71 (1H, *d*, *J* 8.0 Hz, H-5), 6.77 (1H, *d*, *J* 8.0 Hz, H-5′), 6.81 (1H, *dd*, *J* 2.0, 8.0 Hz, H-6), 6.74 (1H, *dd*, *J* 2.0, 8.0 Hz, H-6′), 4.67 (2H, *d*, *J* 5.5 Hz, H-7β, H-7′β), 3.02 (2H, *m,* H-8α, H-8′α), 4.18 (2H, *dd*, *J* 7.0, 14.0 Hz, H-9β, H-9′β), 3.82 (2H, *m*, H-9α, H-9′α), 3.83 (3H, *s*, OCH_3_), 3.80 (3H, *s*, OCH_3_), 5.85 (2H, *s*, OCH_2_O).

(+)-*Eudesmin* (**5**). Yellow oil. 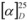 +17.5 ° (CHCl_3_, *c* 0.12) [lit. +61 ° (CHCl_3_, *c* 0.4)] [17]. ^1^H-NMR and ^13^C-NMR data were consistent with those previously reported [17].

*trans*-*N*-Feruloyl tyramine (**6**). Amorphous solid. ^1^H-NMR (DMSO-*d_6_*) δ 6.43 (1H, *d*, *J* 15.9, H-2), 7.31 (1H, *d*, *J* 15.9, H-3), 7.11 (1H, *d*, *J* 1.8, H-2°), 6.79 (1H, *d*, *J* 8.1, H-5′), 6.98 (1H, *dd*, *J* 8.1, 1.8, H-6′), 3.32 (2H, *m*, H-2″), 2.65 (2H, *t*, *J* 7.2, H-3″), 6.68 (2H, *d*, *J* 8.5, H-2′′′, H-6′′′), 7.00 (2H, *d*, *J* 8.5, H-3′′′, H-5′′′), 3.80 (3H, *s*, OCH_3_), 7.95 (1H, *t*, *J* 5.7, NH).

*Aristolactam Ia N-β-D-glucoside* (**7**). Amorphous solid. 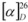
−7.9 ° (MeOH, *c* 0.1) [lit. −9.9 ° (MeOH, *c* 0.07)] [19]. ^1^H-NMR data were consistent with those previously reported [19].

*Aristolactam Ia 8-β-D-glucoside* (**8**). Amorphous solid. 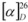
−8.2 ° (MeOH, *c* 0.1) [lit. −10.5 ° (MeOH, *c* 0.2)] [20]. ^1^H-NMR data were consistent with those previously reported [20].

*Aristolactam IIIa* (**9**). Amorphous solid.^ 1^H-NMR (DMSO-*d_6_*) δ 7.62 (1H, *s*, H-2), 7.97 (1H, *d*, *J* 2.5, H-5), 7.10 (1H, *dd*, *J* 2.5, 8.5, H-7), 7.79 (1H, *d*, *J* 8.5, H-8), 7.05 (1H, *s*, H-9), 6.48 (2H, *s*, OCH_2_O), 10.65 (1H, *s*, NH).

*Aristolactam 9-O-β-D-glucopyranosyl-(1→2)-β-D-glucoside* (**10**). Amorphous solid. 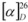
−3.9 ° (*c* 0.5, MeOH). ^1^H-NMR (CDCl_3_) and ^13^C-NMR (CDCl_3_) spectra see Table 1; ESI-HR-TOF-MS (probe), 4000 V, *m/z* (rel. int.): 632. 1614 [M − H]^−^ (100) (calculated for C_29_H_31_O_15_N − H = 632.1615); ESI-MS/MS (probe) 4,500 V from ions at *m/z* 632.1 (100), *m/z* (rel. int.): 308.1 [M − glc-glc]^− ^(92).

*Magnoflorine* (**11**). Amorphous solid. 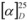
+164.4 ° (MeOH, *c* 0.03) [lit. +150.0 ° (MeOH, *c* 0.1)] [23]. ^1^H-NMR (DMSO-*d_6_*) δ 6.49 (1H, *s*, H-3), 2.7–2.8 (2H, *m*, H-4α, H-4β), 3.7–3.6 (2H, *m*, H-5α, H-5β), 4.34 (1H, *br d*, *J* 13.0, H-6a), 2.59 (1H, *t*, *J* 13.0, H-7α), 3.10 (1H, *br d*, *J* 13.0, H-7β), 6.35 (1H, *br d*, *J* 8.0, H-8), 6.59 (1H, *d*, *J* 8.0, H-9), 3.65 (3H, *s*, OCH_3_-10), 3.68 (3H, *s*, OCH_3_-2), 2.88 (3H, *s*, N-CH_3_), 3.29 (3H, *s*, N-CH_3_). ^13^C-NMR (DMSO-*d_6_*) δ 152.1 (C-1), 151.2 (C-2), 108.8 (C-3), 111.6 (C-3a), 23.2 (C-4), 60.5 (C-5), 69.2 (C-6a), 30.4 (C-7), 125.1 (C-7a), 112.4 (C-8), 109.9 (C-9), 150.3 (C-10), 152.5 (C-11), 122.6 (C-11a), 123.1 (C-1a), 120.0 (C-1b), 55.2, 55.7 (OCH_3_), 42.5, 52.6 (N-CH_3_).

*trans-N-Coumaroyltyramine* (**12**). ^1^H-NMR (DMSO-*d_6_*) δ 6.38 (1H, *d*, *J* 15.9, H-2), 7.30 (1H, *d*, *J* 15.9, H-3), 7.37 (2H, *d*, *J* 8.7, H-2′, H-6′), 6.78 (2H, *d*, *J* 8.7, H-3′, H-5′), 3.31 (2H, *m*, H-2″), 2.64 (2H, *t*, *J* 7.2, H-3″), 7.00 (2H, *d*, *J* 8.4, H-2′′′, H-6′′′), 6.67 (2H, *d*, *J* 8.4, H-3′′′, H-5′′′), 7.94 (1H, *m*, NH).

*trans-N-Feruloyl-3-O-methyldopamine* *(**13**). *^1^H-NMR (DMSO-*d_6_*) δ 6.43 (1H, *d*, *J* 15.3, H-2), 7.30 (1H, *d*, *J* 15.3, H-3), 7.10 (1H, *d*, *J* 2.1, H-2′), 6.78 (1H, *d*, *J* 7.8, H-5′), 6.97 (1H, *dd*, *J* 7.8, 2.1, H-6°), 3.32 (2H, *m*, H-2″), 2.65 (2H, *t*, *J* 6.9, H-3″), 6.77 (1H, *d*, *J* 2.1, H-2′′′), 6.68 (1H, *d*, *J* 7.8, H-5′′′), 6.60 (1H, *dd*, *J* 7.8, 2.1, H-6′′′), 3.79 (3H, *s*, OCH_3_), 3.74 (3H, *s*, OCH_3_), 7.94 (1H, *m*, NH).

*N-cis- and N-trans-p-Coumaroyl-3-O-methyldopamine* (**14** + **15**). Colorless oil. ^1^H-NMR (CDCl_3_) spectra see Table 1. ^13^C-NMR (DMSO-*d_6_*) δ 137.8 (C-3), 127.0 (C-2′, 6′), 116.0 (C-3′, 5′), 113.2 (C-2′′′), 120.9 (C-6′′′), 147.4 (C-3′′′), 145.1 (C-4′′′), 130.6 (C-1′′′), 126.3 (C-1′), 159.0 (C-4′), 56.0 (OCH_3_). ESI-HR-TOF-MS (probe) 4,000V, *m/z* (rel. int.): 312.1233 [M − H]^−^ (100) (calculated for C_18_H_19_O_4_N − H = 312.1236).

## 4. Conclusions

Extracts from different parts of *A. gigantea* showed a diverse chemical composition. As previously observed, the characteristic chemical constituents of the leaves of this species arebisbenzylisoquinolinic and 8-benzylberberinic alkaloids, whereas stems contain lignans at high concentrations, alkamides, and aristolactams. Among the compounds that were isolated from stems, two alkamides and an aristolactam are described here for the first time.
